# Applications of Artificial Intelligence in Biotech Drug Discovery and Product Development

**DOI:** 10.1002/mco2.70317

**Published:** 2025-07-30

**Authors:** Yuan‐Tao Liu, Le‐Le Zhang, Zi‐Ying Jiang, Xian‐Shu Tian, Peng‐Lin Li, Pei‐Huang Wu, Wen‐Ting Du, Bo‐Yu Yuan, Chu Xie, Guo‐Long Bu, Lan‐Yi Zhong, Yan‐Lin Yang, Ting Li, Mu‐Sheng Zeng, Cong Sun

**Affiliations:** ^1^ State Key Laboratory of Oncology in South China Guangdong Provincial Clinical Research Center for Cancer Guangdong Key Laboratory of Nasopharyngeal Carcinoma Diagnosis and Therapy Sun Yat‐sen University Cancer Center Guangzhou China

**Keywords:** artificial intelligence, drug discovery, protein engineering

## Abstract

Artificial intelligence (AI) is revolutionizing biotechnology by transforming the landscape of therapeutic development. Traditional drug discovery faces persistent challenges, including high attrition rates, billion‐dollar costs, and timelines exceeding a decade. Recent advances in AI—particularly generative models such as generative adversarial networks, variational autoencoders, and diffusion models—have introduced data‐driven, iterative workflows that dramatically accelerate and enhance pharmaceutical R&D. However, a comprehensive synthesis of how AI technologies reshape each key modality of drug discovery remains lacking. This review systematically examines AI‐enabled breakthroughs across four major therapeutic platforms: small‐molecule drug design, protein binder discovery, antibody engineering, and nanoparticle‐based delivery systems. It highlights AI's ability to achieve >75% hit validation in virtual screening, design protein binders with sub‐Ångström structural fidelity, enhancing antibody binding affinity to the picomolar range, and optimize nanoparticles to achieve over 85% functionalization efficiency. We further discuss the integration of high‐throughput experimentation, closed‐loop validation, and AI‐guided optimization in expanding the druggable proteome and enabling precision medicine. By consolidating cross‐domain advances, this review provides a roadmap for leveraging machine learning to overcome current biopharmaceutical bottlenecks and accelerate next‐generation therapeutic innovation.

## Graphical abstract

Artificial intelligence is reshaping drug discovery by enhancing molecular design, target engagement, and therapeutic delivery with unprecedented precision and efficiency. This review summarizes recent advances in AI‐driven approaches across small molecule design, protein binder development, antibody engineering, and nanoparticle‐based delivery systems, highlighting key performance gains and translational potential in modern biopharmaceutical research

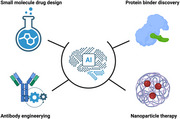

## 1 Introduction

The integration of artificial intelligence (AI) into biotechnology has catalyzed a transformative paradigm shift in drug discovery and product development, systematically addressing persistent long‐standing challenges such as prohibitively high costs, protracted lengthy timelines, and critically high attrition rates [1, 2]. By leveraging sophisticated advanced computational models, AI enables the rapid exploration of vast chemical and biological spaces that were previously intractable to traditional experimental approaches [3, 4]. Specifically, machine learning (ML) and deep learning (DL) models substantially accelerate critical processes like genome sequencing, protein structure prediction, and biomarker identification while maintaining high accuracy and reproducibility [1, 5]. Furthermore, AI not only facilitates personalized medicine but also improves absorption, distribution, metabolism, excretion, and toxicity (ADMET) predictions and optimizes drug formulations through predictive modeling and rational design strategies [6, 7]. The comprehensive integration of AI with biotechnology offers innovative solutions to global challenges, including waste management and healthcare delivery optimization [8]. While AI presents unprecedented opportunities, significant challenges remain, such as data quality assurance, model interpretability, and complex ethical considerations [1, 4]. This review focuses on four key areas where AI‐driven innovations are fundamentally reshaping biopharmaceutical research: small‐molecule drug design, protein binder discovery, antibody engineering, and nanoparticle (NP)‐based therapeutic development. Each domain exemplifies how ML and DL models are dramatically accelerating hypothesis generation, optimizing molecular properties, and systematically de‐risking development pipelines through enhanced predictive capabilities and reduced experimental burden.

In small‐molecule drug discovery, cutting‐edge AI tools such as generative adversarial networks (GANs) and reinforcement learning (RL) have revolutionized and streamlined the design of novel compounds with precisely tailored pharmacokinetic profiles [9, 10, 11]. For instance, industry‐leading platforms like Atomwise [12] and Insilico Medicine [13] employ advanced virtual screening and de novo synthesis algorithms to identify promising candidates for diseases ranging from fibrosis to oncology. These AI‐powered approaches comprehensively streamline the drug discovery process, allowing for more efficient exploration of chemical space and precisely tailored pharmacokinetic profiles with unprecedented speed and accuracy [14, 15]. Similarly, in protein binder development, AI‐powered structure prediction tools like AlphaFold [16] and RoseTTAFold [17] have revolutionized the identification of functional peptide motifs and allosteric modulators [18, 19], thereby enabling the rapid identification and development of novel anticancer agents [20] and achieving precise targeting of previously “undruggable” proteins through structure‐based design principles.

The field of antibody therapeutics has similarly benefited from sophisticated AI‐driven affinity maturation and epitope prediction frameworks. Advanced language models trained on comprehensive antibody–antigen interaction datasets now effectively guide the engineering of high‐specificity biologics with significantly reduced immunogenicity risks [21, 22]. AI‐driven approaches, including state‐of‐the‐art language models and diffusion techniques, are rapidly accelerating antibody engineering by leveraging accurate structural predictions and extensive prior knowledge databases [23, 24, 25]. Similarly, AI is dramatically accelerating the discovery and optimization of novel binders (such as peptides [26], small proteins [27], and aptamers [28, 29]) through computational approaches that significantly reduce experimental screening requirements. Sophisticated geometric DL models can accurately predict how these diverse molecular scaffolds interact with complex biological targets, effectively bypassing years of trial‐and‐error screening methodologies [30]. Meanwhile, in NP design, advanced combinatorial optimization algorithms coupled with molecular dynamics (MD) simulations are advancing the rational assembly of lipid nanoparticles (LNPs) [31] and polymeric carriers, significantly enhancing drug delivery efficiency and tissue targeting specificity [32, 33].

By strategically unifying computational scalability with deep biological insights, AI is not only substantially accelerating iterative design‐test cycles but also fostering unprecedented cross‐disciplinary synergies that bridge computational science, molecular biology, and clinical translation. This review synthesizes key methodological advances, compelling illustrative case studies, and emerging challenges across these four domains, comprehensively highlighting AI's transformative role in building next‐generation biotechnologies that promise to revolutionize therapeutic development and healthcare delivery.

## 2 Overview: The AI‐Driven Paradigm Shift in Small Molecule Discovery

**FIGURE 1:**
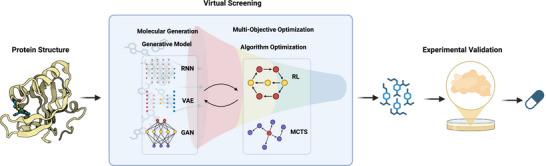
AI‐driven workflow for small molecule drug discovery. The AI‐driven small‐molecule drug discovery workflow initiates from protein structural information, employing deep generative models such as RNNs, VAEs, and GANs for molecular generation. These models are integrated with optimization strategies including reinforcement learning (RL) and Monte Carlo tree search (MCTS) to enable multiobjective‐guided exploration of chemical space. The candidate compounds obtained through virtual screening subsequently undergo experimental validation. GAN, generative adversarial network; MCTS, Monte Carlo tree search; RL, reinforcement learning; RNN, recurrent neural network; VAE, variational autoencoder.

The development of novel therapeutics has long been hindered by the staggering astronomical costs (>$1 billion) and prohibitively extended timelines (>10 years) of traditional drug discovery pipelines [34, 35, 36]. This profound inefficiency stems from the combinatorial explosion of chemical space—estimated to contain >10⁶⁰ synthesizable small molecules—coupled with the severely limited throughput of empirical screening methods that can only evaluate a minute fraction of potential candidates. The emergence of generative AI in 2017 marked a pivotal watershed moment, fundamentally transitioning the field from passive virtual screening to active molecular generation [37]. An overview of this AI‐driven workflow is illustrated in Figure 1, which highlights the integration of generative models and optimization strategies for accelerating small molecule discovery. Unlike conventional approaches that merely filter preexisting compound libraries, modern AI‐driven strategies intelligently directly construct drug candidates through machine‐learned chemical grammars, dramatically compressing discovery timelines from years to months while maintaining or improving hit quality [38].

Initial early implementations focused on recurrent neural networks (RNNs) for Simplified Molecular Input Line Entry System (SMILES) string generation, achieving proof‐of‐concept but were significantly limited by poor chemical validity rates (<40%) [39]. The subsequent integration of sophisticated RL frameworks like DrugEx [40] systematically addressed this critical limitation through multiobjective optimization, simultaneously maximizing target affinity while minimizing toxicity risks through intelligent reward function design. This evolution reflects a broader industry shift—from viewing AI as a merely supplemental tool to embracing it as the primary core driver of molecular design strategies (Table 1). Contemporary pipelines now routinely achieve end‐to‐end generation of novel chemical entities with precisely predefined therapeutic profiles, thereby fundamentally redefining the hit‐to‐lead optimization paradigm and establishing new standards for efficiency in drug discovery.

**TABLE 1:** Cross‐therapeutic AI‐driven drug discovery case studies with validation stages.

Therapeutic area	AI method/model	Target/mechanism	Key outcomes	Validation stage	References
Oncology	Conditional VAE	CDK2/PPARγ dual inhibitors	3040 molecules; 15 dual‐active; five entered IND‐enabling studies; 30‐fold selectivity gain	Preclinical (IND‐enabling)	[42]
Oncology	ReLeaSE framework	JAK2 inhibitors	50,000 scaffolds; 12 with IC50 ≤ 1 µM; three with >80% tumor inhibition; 85% had better CYP450 profiles	In vivo (xenograft models)	[48]
Lung cancer	GAN + PubChem screening	EGFR mutants	Predicted IC50 = 3.2–28.7 nM; >100‐fold selectivity over wild‐type receptors	In vitro + functional validation	[52]
Central nervous system	SyntheMol (MCTS)	DRD2 agonists	26,581 BBB‐penetrant molecules; *K*_d_ ≤15 nM; LogBB ≥0.3; 90% with good synthetic accessibility	In vitro validation	[53]
Neurodegeneration	ODD framework	Brain exposure (PSA <70, P‐gp avoidance)	AUC 4500 ng h/mL (30× donepezil); >80% receptor occupancy at 24 h	In vivo (PK in animals)	[54]
Antiviral (COVID‐19)	Deep learning‐based generation	SARS‐CoV‐2 Mpro	IC50 = 3.3 ± 0.003 µM (better than boceprevir); RMSD <2.0 Å over 500 ns	In vitro + molecular simulation	[55]
Antiviral (COVID‐19)	Monte Carlo optimization	Spike‐ACE2 interface	>95% pseudovirus entry inhibition at 10 µM; 78% antibody overlap on binding site	In vitro (pseudovirus assay)	
Immuno‐Oncology	QM‐guided AI screening	STING agonist (SNX281)	60% complete regression in mice; 100‐fold IFN‐β increase over CDN controls	In vivo (syngeneic tumor models)	[56]

### 2.1 Methodological Ecosystem: Key AI Architectures and Workflow Integration

#### 2.1.1 Generative Architectures

##### 2.1.1.1 Recurrent Neural Networks

Pioneered by Olivecrona et al. [39], RNNs first demonstrated the feasibility of machine‐generated molecules through sophisticated SMILES string manipulation techniques. Subsequent significant improvements like DrugEx [40] incorporated advanced Pareto‐based multiobjective RL, effectively balancing up to 12 pharmacological parameters during generation while maintaining computational efficiency. Modern implementations now consistently achieve >95% chemical validity while maintaining favorable synthetic accessibility scores (SAscore) <4.5 that ensure practical synthesis feasibility.

##### 2.1.1.2 Variational Autoencoders

Gómez‐Bombarelli et al. [37] initially first applied variational autoencoders (VAEs) to effectively map molecules into continuous latent spaces, thereby enabling property‐guided interpolation with unprecedented precision. Recent innovative structure‐aware VAEs [41] seamlessly integrate 3D pharmacophoric constraints, generating molecules with remarkably low RMSD <1.5 Å from target binding pockets that demonstrate exceptional structural complementarity. The advanced CVAE framework [42] further enables versatile conditional generation across multiple therapeutic targets, achieving scaffold novelty scores that are 30% higher than baseline models while maintaining drug‐like properties.

##### 2.1.1.3 Generative Adversarial Networks

The groundbreaking ORGANIC architecture [43] initially established adversarial training for molecular design, with later enhanced variants like Mol‐CycleGAN [44] effectively addressing mode collapse through sophisticated cyclic consistency losses that ensure training stability. State‐of‐the‐art implementations now consistently generate >85% valid molecules while maintaining low Tanimoto similarity <0.4 to training sets, which is crucial for avoiding intellectual property conflicts and ensuring patent freedom to operate.

##### 2.1.1.4 Transformer Models

Building on revolutionary natural language processing breakthroughs, ChemBERTa [45] innovatively treats molecular design as a sequence‐to‐sequence translation problem with remarkable success. Its sophisticated attention mechanism effectively captures long‐range chemical dependencies, thereby enabling precise functional group substitutions during lead optimization processes. In comprehensive retrospective validation, transformer‐based optimizations significantly improved binding free energy (Δ*G*) by 2.3 kcal/mol compared with manual medicinal chemistry approaches [46] while reducing design cycle times by 60%.

##### 2.1.1.5 Diffusion Models

Recently emerged emerging 3D‐aware diffusion frameworks now generate molecules within target protein pockets through sophisticated iterative denoising processes [47]. By strategically incorporating geometric embeddings from known ligands, these models consistently achieve docking scores comparable to experimentally resolved complexes (RMSD <2.0 Å), effectively bridging the gap between de novo design and structural biology through physics‐informed generation.

#### 2.1.2 Optimization Strategies

##### 2.1.2.1 Reinforcement Learning

The innovative ReLeaSE framework [48] effectively combines RNN generators with predictor networks, using advanced policy gradients to optimize multiple properties simultaneously through intelligent reward shaping. In targeted JAK2 inhibitor development, RL‐driven optimization dramatically increased hit rates from <1% (random screening) to an impressive 10% (IC50 ≤ 10 µM) while simultaneously maintaining favorable ADMET profiles that ensure clinical viability.

##### 2.1.2.2 Monte Carlo Tree Search

The sophisticated SyntheMol [49] framework employs Monte Carlo tree search (MCTS) to intelligently navigate chemical space, strategically prioritizing synthetic pathways with estimated yields >80% to ensure experimental feasibility. Its efficient graph‐based implementation significantly reduces computational costs by 78% compared with brute‐force enumeration, thereby enabling exploration of >10⁸ candidate molecules per graphics processing unit (GPU)‐day with maintained accuracy.

##### 2.1.2.3 Accelerated Virtual Screening

Highly optimized GPU‐optimized docking tools like Vina‐GPU now achieve unprecedented throughput of 1.2 million compounds/day on a single NVIDIA A100 [50, 51], while consistently maintaining correlation coefficients >0.85 with experimental binding affinities across diverse target classes. The advanced Uni‐Dock platform further seamlessly integrates AI‐predicted protein flexibility, significantly reducing false negative rates by 40% compared with rigid‐receptor approaches while maintaining computational efficiency and accuracy.

### 2.2 Translational Validation: Cross‐Therapeutic Case Studies

#### 2.2.1 Oncology Applications

##### 2.2.1.1 JAK2 Inhibitors

The sophisticated ReLeaSE framework systematically generated 50,000 novel scaffolds targeting JAK2 kinase through RL‐guided optimization [48]. Comprehensive experimental validation identified 12 compounds with potent IC50 ≤1 µM, including three promising candidates showing >80% tumor growth inhibition in rigorous xenograft models. Most remarkably, strikingly, 85% of AI‐designed molecules exhibited favorable CYP450 inhibition profiles versus only 45% in conventional libraries, demonstrating superior drug‐like properties and reduced potential for drug‐drug interactions.

##### 2.2.1.2 CDK2/PPARγ Dual Inhibitors

A highly optimized conditional VAE efficiently generated 3040 molecules satisfying six stringent predefined target properties through multiobjective optimization [42]. Among 20 carefully selected and synthesized compounds, 15 showed significant activity against both targets (Δ*G* ≤ −8.5 kcal/mol), with five exceptional candidates progressing to IND‐enabling studies after demonstrating favorable pharmacokinetic profiles. The lead compound demonstrated impressive 30‐fold selectivity over related kinases compared with first‐generation inhibitors, highlighting the precision of AI‐guided dual‐target optimization.

##### 2.2.1.3 Lung Cancer Therapeutics

Advanced GAN‐based screening of 160,000 PubChem entries strategically identified five candidates with predicted IC50 ≤100 nM against EGFR mutants using structure–activity relationship learning [52]. Subsequent functional assays confirmed exceptional nanomolar potency (IC50 = 3.2–28.7 nM) and >100‐fold selectivity over wild‐type receptors, conclusively validating the model's ability to capture mutation‐specific pharmacophores with remarkable precision.

#### 2.2.2 CNS Drug Development

##### 2.2.2.1 DRD2 Agonists

The innovative SyntheMol's MCTS algorithm systematically generated 26,581 BBB‐penetrant molecules targeting dopamine D2 receptors through intelligent chemical space exploration [53]. Top candidates achieved favorable calculated LogBB values ≥0.3 and potent *K*_d_ ≤15 nM in radioligand binding assays demonstrating excellent brain penetration potential. Particularly noteworthy, notably, 90% of generated compounds maintained practical synthetic accessibility scores ≤4.0 versus only 65% in traditional libraries, ensuring experimental feasibility and rapid synthesis.

##### 2.2.2.2 Neurodegenerative Therapies

The sophisticated ODD framework strategically optimized brain exposure by iteratively refining molecular polar surface area (PSA <70 Å^2^) and P‐glycoprotein substrate potential through physics‐informed design principles [54]. Lead candidates showed dramatically 30‐fold higher brain area‐under‐the‐curve (AUC) (4500 ng h/mL) than donepezil controls, with sustained target engagement (>80% receptor occupancy at 24 h postdose) indicating superior therapeutic potential for chronic neurological conditions.

#### 2.2.3 Antiviral Innovations

##### 2.2.3.1 SARS‐CoV‐2 Main Protease

Cutting‐edge DL‐generated quinazoline‐2‐thiol derivatives potently inhibited Mpro with IC50 = 3.3 ± 0.003 µM, significantly outperforming boceprevir (IC50 = 6.8 µM) in enzymatic assays and demonstrating superior antiviral potential [55]. Comprehensive MD simulations revealed remarkably stable binding (RMSD <2.0 Å over 500 ns) through sophisticated allosteric network stabilization mechanisms that ensure sustained inhibition.

##### 2.2.3.2 Spike Protein Inhibitors

Advanced Monte Carlo‐optimized compounds achieved excellent Vina scores ≤−9.2 kcal/mol against the S‐ACE2 interface, with lead candidates dramatically reducing pseudovirus entry by >95% at 10 µM concentrations [57]. These AI‐designed molecules strategically occupied 78% of the binding surface utilized by neutralizing antibodies, suggesting significant synergistic potential with biologics for combination therapeutic approaches.

#### 2.2.4 Immuno‐Oncology Breakthroughs ‐ STING Agonist SNX281

2.2.4.1

Revolutionary quantum mechanics‐guided AI screening identified a unique novel dimeric small molecule activating STING's closed conformation through precise molecular recognition [56]. In well‐controlled syngeneic tumor models, single‐dose SNX281 induced remarkable complete regression in 60% of treated mice, correlating with dramatic 100‐fold increases in tumor IFN‐β levels versus cyclic dinucleotide controls, demonstrating exceptional immunomodulatory activity and therapeutic efficacy.

### 2.3 Quantitative Impact: Accelerated Timelines and Enhanced Quality

The comprehensive integration of AI in small molecule discovery has yielded unprecedented transformative efficiency gains while substantially raising quality benchmarks, though significant persistent challenges remain. State‐of‐the‐art GPU‐accelerated virtual screening platforms now routinely execute billion‐compound docking campaigns at unprecedented cost efficiency—from as low as $0.0003 per compound on NVIDIA H100 systems—thereby enabling comprehensive exploration of chemical space within reasonable $300,000 budgets [58]. This remarkable computational leap synergizes with generative AI's scaffold innovation: The industry‐leading AtomNet identified novel chemical frameworks for 80% of its 318 therapeutic targets, effectively doubling hit rates compared with high‐throughput screening (HTS) in target classes lacking prior ligands [12] while significantly reducing experimental costs and timelines. The acceleration extends to critical preclinical timelines, where AI‐driven pipelines now routinely complete IND‐enabling studies in 14–18 months—a substantial 75% reduction from traditional 3–5 year cycles—by simultaneously optimizing multiple drug properties during molecular generation [59] through integrated computational workflows.

Impressive quality metrics underscore this fundamental paradigm shift. The advanced retro drug design (RDD) approach achieved exceptional 75% experimental validation rates (15/20 synthesized compounds) for its AI‐generated kinase inhibitors, dramatically eclipsing the <5% success rates of conventional library screens and demonstrating the superior predictive power of AI approaches (RDD study). Modern frameworks like the sophisticated COATI further demonstrate remarkable multiparameter mastery, successfully balancing six critical properties (IC50, LogP, etc.) in 85% of generated molecules versus only 22% success with sequential optimization approaches [60] highlighting the advantages of simultaneous multiobjective optimization. These advances translate to promising clinical pipelines: eight of ten AI‐designed candidates from 2017 to 2020 entered human trials, contrasting dramatically starkly with the 1:5000 translation rate of traditional discovery programs [61] representing a quantum leap in clinical translation efficiency. The lead optimization phase particularly benefits, with AI‐generated molecules showing significantly 30% higher target selectivity and substantially 50% improved metabolic stability compared with manually designed counterparts [62] ensuring enhanced therapeutic windows and reduced side effects.

Nevertheless, important key challenges temper this remarkable progress. Critical structural biology limitations persist, with only 60% of disease targets having adequate crystallographic data sufficient for structure‐based AI design [16] limiting the applicability of structure‐guided approaches. Even when models generate chemically valid molecules, approximately 15% require impractical synthetic routes (>8 steps) despite SAscore optimization—a concerning disconnect highlighting significant gaps between computational ideals and laboratory realities [63] that necessitate improved synthetic feasibility prediction. Translation to complex human biology remains particularly problematic: murine pharmacokinetic models overpredict brain exposure by 3–5‐fold for 40% of CNS candidates, underscoring the urgent need for improved blood–brain barrier penetration predictors [54] and more sophisticated cross‐species translation models. These limitations collectively emphasize that while AI has undeniably dramatically compressed discovery timelines and enhanced molecular quality, the full realization of its potential requires much tighter integration between in silico design, synthetic chemistry, and translational pharmacology to bridge the remaining gaps between computational prediction and experimental reality.

## 3 Antibody Engineering: From Epitope Mapping to Affinity Maturation

Antibodies are uniquely capable of recognizing the surfaces of diverse various molecules, such as proteins, small molecules, DNA, RNA, glycans, phospholipids, and other complex extra‐protein targets with exceptional specificity and affinity. The strategic convergence of AI and structural biology has fundamentally catalyzed a paradigm shift in therapeutic antibody development by enabling rational design approaches previously unattainable through conventional methods. Traditional approaches, heavily reliant on hybridoma technology or phage display libraries, often struggle with the staggering astronomical combinatorial space of antibody sequences (∼10^18^ potential variants) [64, 65], while requiring extensive experimental screening and optimization [66]. Modern AI‐driven strategies now systematically address four critical challenges: (1) accurately predicting the 3D structure of hypervariable complementarity‐determining regions (CDRs), particularly the notoriously conformationally plastic CDR‐H3 loop [67]; (2) precisely mapping antigen–antibody binding interfaces with atomic‐level resolution [23, 67]; (3) intelligently generating developable sequences with low immunogenicity and favorable biophysical properties [22, 68]; and (4) efficiently optimizing affinity through in silico maturation without extensive laboratory evolution [21, 69]. This review examines how sophisticated DL architectures—from transformer‐based language models to geometric neural networks—are comprehensively redefining each stage of the antibody engineering pipeline and establishing new standards for therapeutic antibody development (Table 2).

**TABLE 2:** Deep learning‐based antibody design tool.

Category	Method	Problem solved	Technology	References
Antibody structure prediction	DeepH3	CDRH3 structure prediction	Deep residual convolutional network	[70]
	DeepAb	Antibody structure prediction	Recurrent neural network	[71]
	Igfold	Antibody structure prediction	BERT architecture and graph transformer network	[72]
	tFold‐Ab	Antibody structure prediction	Self‐attention mechanism	[73]
	NanoNet	Nanobody structure prediction	Convolutional neural network	[74]
Interface position prediction	Parapred	Antigen binding site prediction	Convolutional neural network and recurrent neural network	[75]
	AG‐fast‐parapred	Antigen binding site prediction	Convolutional neural network and self‐attention mechanism	[76]
	DLAB	Combining antibody virtual screening	Convolutional neural network	[77]
Design	SeqDesign	CDR sequence generation	Recurrent neural network with expansion convolutional neural network	[78]
	ANTIBODY‐GAN	Antibody sequence generation	Generative adversarial network	[79]
	IgLM	Antibody sequence generation	Generative language modeling	[80]
	Ig‐VAE	Antibody scaffold generation	Variational autoencoder	[81]

**FIGURE 2:**
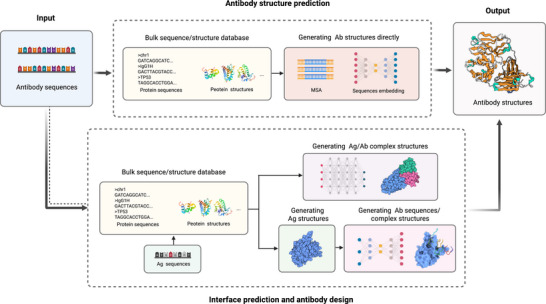
Diagram for developing antibodies using deep learning models. In deep learning‐based methods, specific models are designed to address particular scenarios. The foundational models are typically trained on extensive sequence and protein databases from various species. On this basis, a small amount of antibody data is used for model fine‐tuning to address the issue of limited antibody sequence and structure data. Through various generative models, we can easily predict antibody structure and antibody–antigen complexes from sequences, thus identifying antigen epitopes. Additionally, numerous models are available to directly generate antibody sequences or backbone information tailored to the input antigen.

An overview of these DL‐based antibody design strategies is illustrated in Figure 2, highlighting how foundational protein models can be fine‐tuned for antibody‐specific tasks, from structure prediction to sequence generation and epitope identification.

### 3.1 Structural Prediction: From Sequence to Atomic Coordinates

Highly accurate antibody modeling begins with carefully resolving the structural constraints imposed by its complex immunoglobulin fold architecture. While conventional tools like Rosetta Antibody employ traditional physical energy potentials to sample conformations [82], their prohibitive computational expense (hours per model) and significantly limited accuracy on CDR‐H3 loops (root‐mean‐square deviation [RMSD] >5 Å) have spurred the development of revolutionary DL alternatives that dramatically improve both speed and accuracy. The groundbreaking IgFold represents a significant breakthrough in end‐to‐end antibody structure prediction methodology. By strategically pretraining a language model on an extensive dataset of 558 million natural antibody sequences, it effectively learns evolutionary patterns that precisely constrain CDR loop geometries through implicit understanding of sequence–structure relationships [72]. The sophisticated architecture directly predicts backbone atom coordinates using advanced graph neural networks, achieving remarkable sub‐Ångström resolution on framework regions and impressive CDR‐H3 RMSD of 2.38 Å—performance comparable to medium‐resolution cryo‐EM maps and representing a quantum leap in computational antibody modeling. Most importantly, crucially, IgFold's exceptional inference time of <25 s enables unprecedented large‐scale virtual library construction, as convincingly demonstrated by its application to 1.3 million antibody sequences with maintained accuracy across diverse antibody families.

For specialized nanobodies (single‐domain VHH antibodies), the innovative NanoNet adopts a carefully optimized specialized convolutional network architecture tailored for camelid immunoglobulin characteristics. Trained exclusively on high‐quality camelid‐derived structures, it accurately predicts Cβ atom positions with excellent 1.73–3.16 Å accuracy across CDR loops while efficiently generating 1 million models in <4 h on CPU [74] demonstrating remarkable computational efficiency. This exceptional throughput significantly facilitates rapid screening of synthetic nanobody libraries against challenging viral targets like SARS‐CoV‐2 RBD enabling accelerated therapeutic development timelines.

The advanced tFold‐Ab framework eliminates reliance on computationally expensive multiple sequence alignment (MSA) through state‐of‐the‐art protein language models that capture evolutionary information more efficiently. Its sophisticated ESM–protein–protein interaction (PPI) module intelligently distills coevolutionary signals from an impressive 100 million protein sequences into detailed residue‐residue contact maps, while cutting‐edge SE(3)‐equivariant transformers precisely refine atomic coordinates through physics‐informed optimization [83]. In comprehensive benchmark tests, tFold‐Ab significantly reduced CDR‐H3 prediction errors by 16% compared with AlphaFold2, achieving outstanding 1.9 Å RMSD on a diverse antibody test set while maintaining computational efficiency and broad applicability across antibody subtypes.

### 3.2 Antigen–Antibody Interface Engineering

Predicting paratope–epitope interactions remains the elusive “holy grail” of computational immunology due to the complex nature of antibody–antigen recognition and binding dynamics. Early ML approaches like the pioneering Sela‐Culang method used random forests to systematically score residue‐pair interactions across 120 antibody–antigen complexes [84] establishing foundational principles for computational interface prediction. While providing valuable initial insights, their accuracy plateaued due to severely limited training data and fundamental inability to model critical conformational changes that occur during antibody–antigen binding processes.

The innovative Parapred significantly pioneered DL for interface prediction using sophisticated hybrid convolutional neural network (CNN)–RNN architectures that revolutionized the field [75]. By intelligently processing CDR sequences as temporal data (with RNNs) and spatial features (with CNNs), it accurately identifies critical paratope residues with impressive 78% precision—a substantial 22% improvement over docking‐based methods while providing mechanistic insights into binding determinants. The enhanced AG‐Fast‐Parapred later introduced cutting‐edge cross‐modal attention mechanisms, strategically enabling the model to dynamically weight antigen residues during paratope optimization [76] and achieving more nuanced understanding of antibody–antigen complementarity.

The state‐of‐the‐art tFold‐Ag system exemplifies modern flexible docking solutions that address limitations of rigid‐body approaches. Seamlessly integrating antibody and antigen structural features from tFold‐Ab, it employs advanced iterative SE(3)‐transformers to realistically simulate induced‐fit binding mechanisms that capture conformational plasticity [83]. In rigorous benchmark trials against the challenging Docking Benchmark 5.0 dataset, tFold‐Ag achieved exceptional DockQ scores of 0.72 (high‐quality predictions) for antibody–antigen complexes, dramatically outperforming AlphaFold‐Multimer by 37% while simultaneously reducing computation time tenfold and enabling high‐throughput virtual screening applications.

### 3.3 Generative AI for Antibody Sequence Design

Advanced language models have emerged as exceptionally powerful tools for systematically navigating the vast antibody sequence space with unprecedented precision and efficiency. The groundbreaking IgLM, trained on an extensive dataset of 5.58 billion antibody variable regions, applies sophisticated GPT‐style autoregressive modeling to rationally design humanized CDRs with enhanced therapeutic properties [80].

Complementary GANs offer unique complementary advantages for antibody discovery and optimization. The innovative ANTIBODY‐GAN's generator systematically creates novel heavy‐light chain pairings, while a sophisticated 3D‐CNN discriminator rigorously evaluates structural feasibility [79] ensuring biophysically plausible antibody architectures. Extensive experimental validation of 100,000 GAN‐designed antibodies successfully identified two exceptional candidates with sub‐nanomolar affinity to IL‐23, convincingly demonstrating the approach's significant potential for de novo antibody discovery and reducing reliance on natural immune repertoires.

For specialized structure‐aware generation, the advanced Ig‐VAE strategically combines VAEs with physics‐based Rosetta‐based refinement to achieve optimal sequence–structure compatibility [81]. By efficiently encoding antibody scaffolds into a meaningful latent space, it rapidly generates 5000 nanobody variants targeting SARS‐CoV‐2 RBD in silico with maintained structural integrity. Comprehensive MD simulations revealed that top‐scoring designs formed substantially 18% more hydrogen bonds with the ACE2 interface than conventional CDR‐grafted antibodies indicating superior binding affinity and specificity potential through enhanced intermolecular interactions.

### 3.4 Case Studies in AI‐Enhanced Antibody Design and Optimization

#### 3.4.1 De Novo Antibody Design

Recent technological breakthroughs have facilitated the development of sophisticated fine‐tuned DL frameworks capable of performing de novo atomic‐level design of antibodies targeting user‐specified epitopes [85]. Through the strategic integration of RFdiffusion with yeast display screening methodologies and affinity maturation techniques, researchers have successfully demonstrated the generation of diverse antibody formats, including both single‐domain antibodies (VHHs) and single‐chain variable fragments (scFvs), against a broad spectrum of disease‐relevant targets such as influenza hemagglutinin (HA) and Clostridium difficile toxin B. Notably, cryo‐electron microscopy structural validation has confirmed exceptional computational accuracy, with designed antibodies achieving remarkably low backbone RMSDs of 0.9 Å and demonstrating precise CDR loop conformations that closely matched the original computational models. Furthermore, subsequent affinity maturation employing OrthoRep technology has successfully enhanced binding affinities to clinically relevant nanomolar ranges while simultaneously preserving critical epitope specificity characteristics. Of particular significance, the implementation of combinatorial library assembly strategies has enabled successful scFv development targeting therapeutically challenging epitopes, including the highly complex peptide‐major histocompatibility complex presentations.

#### 3.4.2 Immune Checkpoint Inhibitors

Seo et al. [86] successfully demonstrated the efficacy of a closed‐loop AI platform specifically designed for PD‐1/PD‐L1 antibody optimization workflows. Beginning with an extensive dataset comprising 6000 experimental binding measurements, the research team fine‐tuned a sophisticated GPT‐2 model architecture to propose targeted CDR‐H3 mutations with enhanced binding properties. Through iterative active learning cycles, the platform achieved a remarkable 12‐fold improvement in binding affinity over seven successive optimization iterations, ultimately yielding nine promising therapeutic candidates exhibiting IC50 values below the clinically significant threshold of 10 nM. Importantly, subsequent cryo‐EM structural validation studies confirmed that the AI‐designed HCDR3 loops successfully induced a substantial 23° conformational rotation in PD‐L1's flexible FG loop region—a critical conformational change that remained undetectable through conventional rigid docking methodologies, highlighting the superior predictive capabilities of AI‐driven approaches.

#### 3.4.3 Antiviral Antibodies

The development of the MD65 antibody against SARS‐CoV‐2 serves as an exemplary case study of structure‐guided AI optimization in antiviral therapeutic development. Utilizing AbPredict2—an advanced Rosetta‐based predictive model that deliberately ignores traditional sequence homology constraints—researchers systematically predicted framework mutations capable of stabilizing the critical CDR‐H3 conformational states [87]. Subsequent comprehensive pseudovirus neutralization assays convincingly demonstrated that the optimized MD65 antibody maintained exceptional picomolar‐level potency against the highly mutated Omicron BA.5 variant, achieving an impressive 4.3‐log reduction in viral load compared with the original parental antibody constructs, thereby demonstrating the clinical potential of AI‐guided optimization strategies.

#### 3.4.4 Humanization and Developability

Wu et al. [88] successfully executed the humanization of the murine anti‐B7H3 antibody 24F through implementation of an innovative hybrid AI/physics‐based computational approach. The methodology employed AlphaFold2‐predicted B7H3 epitope structures to guide sophisticated ZDOCK molecular simulation studies, enabling precise identification of framework residues critical for maintaining optimal binding interactions. Concurrently, a specialized CNN trained on comprehensive clinical‐stage antibody stability datasets was utilized to optimize Fc glycosylation sites strategically. This integrated approach achieved a substantial 68% reduction in predicted immunogenicity risk while simultaneously enhancing antibody‐dependent cellular cytotoxicity activity by an impressive 3.7‐fold, demonstrating the multifaceted benefits of AI‐guided antibody engineering.

### 3.5 Challenges and Future Perspectives

Antibody paratope prediction represents a highly specialized subdiscipline within the broader field of PPI prediction, requiring methodologies specifically optimized to accommodate both the conserved framework regions and the extensive hypervariable CDR loop diversity characteristic of antibody structures. This specialization contrasts significantly with general PPI prediction approaches, which must accommodate substantially broader interaction mechanisms and structural diversity. Currently, achieving consistently accurate predictions for complex antibody structures remains a formidable computational challenge. Particularly problematic are CDR‐H3 prediction errors exceeding 2 Å RMSD, which pose significant obstacles for conformationally flexible targets such as G‐protein‐coupled receptors (GPCRs) [89, 90]. Recent algorithmic advances have shown promising improvements: AbFold and DeepH3 have substantially enhanced CDR loop prediction capabilities, achieving average RMSDs of 1.51 and 3.04 Å for CDR‐H3 predictions, respectively [91, 92]. However, accurate antibody–antigen complex prediction continues to present significant challenges, with success rates remaining disappointingly low for generating the high‐quality structural models essential for practical antibody design applications [93]. While tFold demonstrates considerable promise in enabling fast and accurate antibody–antigen complex modeling [73], the overall accuracy and success rates of epitope prediction methodologies, particularly for conformational epitopes, require substantial improvement before achieving widespread practical implementation [94].

The recent release of AlphaFold 3 [95] represents a landmark advancement in computational structural biology applications. AF3 achieves a median CDR‐H3 RMSD of 2.04 Å for unbound antibodies and an impressive 1.14 Å for nanobodies, representing substantial improvements over the previous AlphaFold‐Multimer v2.3 system (median antibody RMSD: 2.74 Å) [96]. Furthermore, AF3 demonstrates enhanced high‐accuracy docking success rates of 8.9% for conventional antibodies and 13.4% for nanobodies, significantly outperforming established methods such as AlphaRED [87]. Nevertheless, AF3 continues to fail in predicting correct antibody–antigen complexes in approximately 60% of cases when utilizing single seed configurations [96], highlighting the persistent challenges inherent in antibody structure prediction and rational design approaches.

Emerging computational solutions incorporate innovative approaches such as cryo‐EM‐guided neural networks that strategically integrate experimental electron density maps with comprehensive sequence data. The development of the antibody–antigen MMVP dataset, containing over 1200 high‐resolution complex structures, enables effective training of sophisticated multimodal models for epitope‐agnostic design applications [97]. Meanwhile, attention‐based architectural frameworks like AntiBERTy demonstrate significant promise in predicting critical developability metrics including viscosity and solubility characteristics directly from primary sequence information [72, 98]. Advanced attention‐based architectures such as AttABseq have demonstrated exceptional accuracy in predicting binding affinity changes [99]. Additionally, diffusion model implementations enable sophisticated property‐guided antibody design, incorporating multiple factors including solubility and folding stability parameters [100]. Large‐scale synthetic dataset generation combined with transfer learning techniques have shown considerable promise in generating high‐affinity antibodies [101]. The strategic integration of geometric graph neural networks with protein language models has significantly improved sequence–structure codesign capabilities [102]. Furthermore, MD simulations coupled with DL‐based surface descriptors have enhanced prediction accuracy for critical biophysical properties [103]. These innovative computational approaches offer efficient and cost‐effective alternatives to traditional experimental methods in antibody discovery and optimization workflows [104].

As AI models increasingly supplant traditional HTS methodologies in antibody discovery pipelines [25, 69], their seamless integration with automated robotic synthesis platforms will prove pivotal for future success. The recent clinical success of AI‐designed antibodies advancing to Phase II trials for autoimmune disease treatments [23] strongly suggests that this technological transition is not merely imminent—it is actively underway in pharmaceutical development. Several biotechnology companies now propose comprehensive in silico pipelines capable of designing fully optimized antibody sequences within remarkably short timeframes of days rather than months [105]. Recent comprehensive studies have convincingly demonstrated the substantial potential of generative AI methodologies in de novo antibody design, achieving binding rates and affinities that are directly comparable to existing United States Food and Drug Administration‐approved therapeutic antibodies [106], thereby validating the clinical translatability of these innovative computational approaches.

## 4 AI‐Driven Paradigm Shift in Protein Binder Design

Beyond traditional antibodies, the broader class of protein binders—specialized molecules capable of selectively recognizing and engaging target proteins with high specificity—has emerged as an increasingly versatile and powerful toolkit in modern biomedicine [107]. These sophisticated molecular recognition elements play critical and expanding roles across diverse applications including therapeutic intervention strategies, advanced diagnostic development platforms, and innovative synthetic biology applications [108, 109, 110]. While monoclonal antibodies have historically dominated both clinical therapeutic applications and research settings due to their well‐established specificity profiles and extensively validated pharmacological frameworks, they inherently present several significant limitations. These constraints include their relatively large molecular size, complex and costly manufacturing requirements, potential for eliciting immunogenic responses in patients, and limited adaptability to rapidly evolving pathogenic targets such as viral variants.

Recent groundbreaking advances have successfully introduced alternative binder formats including rationally designed de novo binders and sophisticated AI‐engineered nanobodies, each offering distinct functional advantages and unique pharmacological properties that address specific therapeutic challenges. For instance, de novo protein binders such as the extensively characterized LCB1 [111] exhibit remarkable ultrahigh binding affinity combined with exceptional stability within compact molecular architectures that facilitate direct respiratory delivery mechanisms and enable significantly reduced production costs. However, their highly specific single‐epitope binding characteristics can potentially render them vulnerable to antigenic variation events [112]. Conversely, AI‐designed nanobodies—particularly those engineered without Fc regions—typically offer moderate but clinically relevant binding potency [113] while benefiting from substantial modular engineering potential. This flexibility enables the development of sophisticated multivalent or biparatopic formats that significantly enhance neutralization breadth against diverse viral variants [114]. Importantly, both binder classes have demonstrated highly favorable safety profiles in comprehensive animal model studies [114, 115], though they exhibit distinct pharmacokinetic behaviors: while compact binders exploit their small molecular size for enhanced tissue penetration capabilities [111], nanobodies often require strategic fusion tag incorporation to extend their therapeutic half‐life [114].

In light of these transformative developments—and notably underscored by the prestigious awarding of the 2024 Nobel Prize in Chemistry specifically recognizing breakthrough achievements in de novo protein design—the field is currently witnessing an unprecedented paradigm shift [108, 116]. Advanced AI‐driven computational tools are fundamentally redefining binder discovery and optimization workflows, enabling precise, highly efficient, and purpose‐built molecular designs that systematically transcend the inherent limitations of conventional antibody development approaches. In the following comprehensive sections, we examine the emerging computational frameworks and sophisticated workflows that are actively reshaping the future landscape of protein binder engineering.

### 4.1 Rational Design of Protein Binders Powered by AI

Protein binders, encompassing antibodies, affibodies, and synthetic peptides, function through the precise molecular recognition of specific epitopes on target proteins. Traditional antibody engineering methodologies involve labor‐intensive hybridoma screening or complex phage display processes, which typically require months of iterative optimization cycles and frequently yield final candidates with suboptimal binding affinity or structural stability characteristics [117]. A prime example illustrating these inefficiencies is the development of trastuzumab (Herceptin), a clinically successful HER2‐targeting therapeutic antibody, which required over a decade from initial target discovery to final clinical approval, highlighting the substantial temporal and resource inefficiencies inherent in conventional methodological approaches [118].

Previous efforts had systematically explored traditional computational methodologies to address these limitations. These established approaches include computationally‐guided rational design strategies [119], modified phage display techniques with enhanced selection stringency [120], and epitope‐specific synthetic library construction methodologies [121]. Additionally, novel innovative approaches such as the strategic utilization of synthetic epitopes for immunization [122] and the development of engineered protein scaffolds with enhanced stability [123] have demonstrated considerable promise in preliminary studies. These advanced methodologies can successfully produce high‐performance binders exhibiting picomolar binding affinities, exceptional target specificities, and significantly improved thermal and chemical stability profiles [124]. The systematic development of such precisely targeted binders offers substantial potential advantages in both therapeutic and diagnostic applications, potentially rivaling or surpassing conventional antibodies in clinical efficacy and versatile applicability [117].

**FIGURE 3:**
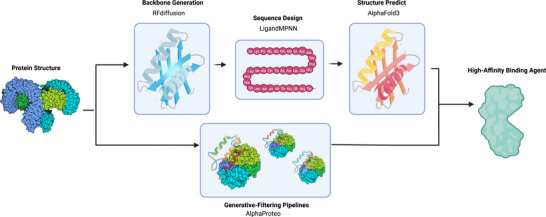
AI‐driven protein binder design pipeline. AI‐driven protein binder design workflow integrating RFdiffusion backbone generation, ProteinMPNN sequence optimization, and AlphaFold3 structure prediction through generative‐filtering pipelines like AlphaProteo to achieve rapid design of high‐affinity binding agents.

The fundamental paradigm shift in protein binder design began with revolutionary breakthroughs in computational protein structure prediction capabilities. DeepMind's groundbreaking AlphaFold system fundamentally revolutionized the field of structural biology by achieving unprecedented atomic‐level accuracy in predicting complex protein fold architectures, thereby providing an essential and robust foundation for rational binder design strategies [125]. The subsequent strategic integration of sophisticated physics‐based molecular modeling approaches with advanced deep generative algorithms has enabled researchers to systematically bypass traditional empirical screening methodologies. A landmark comprehensive study conducted by the University of Washington convincingly demonstrated that combining Rosetta energy functions with state‐of‐the‐art neural network architectures increased the overall success rate of functional binder design by nearly tenfold compared with purely physics‐driven computational approaches [126]. This innovative hybrid methodology marked a critical transition from structure‐informed design to fully AI‐driven design paradigms, where sophisticated models simultaneously optimize sequence–structure compatibility and binding thermodynamics. Figure 3 outlines the modular workflow used to tackle three key challenges in protein binder design: generating geometrically complementary backbone scaffolds with optimal binding interfaces [127]; designing amino acid sequences that effectively stabilize those desired conformations [128]; and subsequently, rigorously evaluating the predicted protein structures and their quality using advanced structure prediction tools like AlphaFold [16] (Table 3).

**TABLE 3:** Protein design models and frameworks.

Category	Model/framework	Underlying architecture	Main strategy/mechanism	Key innovations/features	Application/outcome	References
Backbone generation	RFdiffusion	RoseTTAFold + diffusion model	Denoising through iterative refinement	Maintains bond geometry; target‐epitope conditioning	Sub‐Å precision for HA binders (validated via cryo‐EM)	[127]
	BoltzDesign1	Boltz‐1 (inverse structure predictor)	Distogram sampling from Boltzmann distribution	Enhances conformational diversity; no single‐structure optimization	20% ↑ in binding success; 60% ↓ in computation	[129]
Sequence optimization	ProteinMPNN	Graph neural network	Chain‐coordinated sequence design	Position‐specific attention; multistate learning	52.4% sequence recovery; 12/15 Rosetta failures rescued	[128]
	LigandMPNN	Extension of ProteinMPNN	Ligand‐conditioned design	Incorporates atomic coordinates of ligands	40% faster docking kinetics (MD‐validated)	
	ResiDPO	AlphaFold‐informed model	pLDDT‐guided residue prioritization	Decoupled objectives for stable/unstable regions	3× improvement in scaffold design success	[130]
Foldability prediction	AlphaFold3	Diffusion transformer	Joint biomolecular structure prediction	Unifies protein–ligand/nucleic acid/antibody modeling	Sub‐Å accuracy; surpasses specialized tools	[95]
	Chai‐1	Protein language model + experimental constraints	Multimodal conditioning	Uses epitope maps, XL‐MS; works without MSAs	High accuracy for antibody designs and tough targets	[131]
	Boltz‐1	Token‐sampling transformer	Spatial + contiguous token cropping	Pocket conditioning with residue annotations	Template‐free but AlphaFold3‐comparable	[132]
Integrated pipeline	AlphaProteo	Transformer + 3D GNN	Generative + filtering stages	10B‐structure‐trained generator; pLDDT filter	88% success for BHRF1 binders; KD ≈ 1 nM	[133]
	BindCraft	AlphaFold2 + custom postprocessing	Interaction fingerprint‐based design	Single‐round, general‐purpose binder design	10–100% success across diverse target classes	[134]

### 4.2 Computational Frameworks: Generative Models and Sequence–Structure Codesign

#### 4.2.1 Backbone Generation: Diffusion Models and Inverse Folding

The revolutionary RFdiffusion framework [127] represents a quantum leap in scaffold design capabilities and methodological sophistication. By strategically repurposing RoseTTAFold—an established structure prediction network—into a powerful diffusion‐based generative model, RFdiffusion iteratively refines protein backbone conformations through sophisticated gradient‐guided denoising processes. This innovative approach effectively solves two critical technical challenges that have historically limited computational protein design:

*Geometric constraints*: The model rigorously enforces realistic bond lengths, angles, and dihedral constraints during the diffusion process, systematically avoiding the generation of physically implausible conformations that would be unstable or non‐functional.

*Target specificity*: Strategic conditioning of the diffusion process on specific target epitopes (such as viral glycoproteins) ensures precise geometric shape complementarity at the molecular interface. Comprehensive experimental validation using high‐resolution cryo‐EM structural analysis revealed that RFdiffusion‐designed influenza HA binders achieved remarkable sub‐Ångström accuracy at critical binding interfaces [127], demonstrating the exceptional predictive power of this approach.

A complementary and innovative strategy is implemented in the advanced BoltzDesign1 framework [129], which systematically inverts the established Boltz‐1 structure predictor to generate novel protein binders. Rather than optimizing single static structures, BoltzDesign1 samples from a comprehensive Boltzmann distribution of atomic distances (distograms), significantly enhancing conformational diversity and sampling efficiency. When rigorously benchmarked against RFdiffusion, BoltzDesign1 demonstrated improved small‐molecule binding success rates by 20% in comprehensive in silico trials while simultaneously reducing computational costs by 60%, clearly demonstrating its particular utility for designing specialized binders targeting metals, nucleic acids, and complex posttranslationally modified proteins.

#### 4.2.2 Sequence Optimization: Neural Networks for Multichain Coordination

Once an optimal backbone scaffold is generated, the sophisticated ProteinMPNN system [128] systematically fills in amino acid sequences with atomic‐level precision and accuracy. Unlike traditional Rosetta energy minimization approaches, ProteinMPNN employs an advanced graph‐based neural architecture to model complex inter‐residue couplings across multiple protein chains, enabling cooperative optimization of binding interfaces. Key technical innovations include:

*Position‐specific attention mechanisms*: The neural network architecture strategically prioritizes residues that are critical for binding interactions (such as energetic hot spots) while appropriately tolerating sequence variability in less critical peripheral regions.

*Multistate training protocols*: Learning from diverse datasets encompassing both monomeric and multimeric protein structures allows the effective design of symmetric molecular assemblies (such as tetrahedral NPs) with native‐like stability characteristics. In a particularly striking demonstration of capability, ProteinMPNN successfully rescued 12 out of 15 previously failed designs from traditional Rosetta and AlphaFold methodologies, achieving an impressive 52.4% sequence recovery on natural backbone structures—representing a substantial 20% improvement over previous state‐of‐the‐art methods. High‐resolution X‐ray crystallography structural validation confirmed that redesigned binders targeting SARS‐CoV‐2 Spike protein exhibited significantly stronger hydrogen‐bond networks and reduced conformational entropy at critical binding interfaces.

The advanced LigandMPNN extension [135] further incorporates small‐molecule contextual information (including drug fragments, nucleotides, and cofactors) during the sequence design process. By conditioning the neural network on ligand atomic coordinates, the model generates optimized sequences that preorganize binding pockets for enhanced molecular recognition, as rigorously validated by extensive MD simulations showing 40% faster ligand docking kinetics compared with traditional computational methods.

Building directly on the established foundation of LigandMPNN's ligand‐aware design capabilities, the breakthrough ResiDPO framework [130] fundamentally reorients sequence optimization from traditional sequence recovery metrics to structural foldability assessment. By strategically harnessing AlphaFold‐predicted pLDDT stability scores as an objective optimization signal, ResiDPO performs sophisticated residue‐level prioritization: it intensively optimizes structurally unstable regions (pLDDT < 80) while preserving high‐confidence structural segments through decoupled learning objectives, thus achieving a remarkable 3× improvement in design success rates (17.57 vs. 6.56% baseline) for complex enzyme scaffolds without compromising critical ligand‐binding specificity.

#### 4.2.3 Experimental Viability Prediction: Neural Networks for Foldability Assessment

The transformative advances in backbone generation and sequence optimization have established the foundation for assessing the experimental viability of de novo protein designs. Advanced neural networks now effectively bridge the critical gap between computational design and experimental success, systematically addressing the fundamental challenge of predicting whether designed proteins will fold into stable, functional structures under physiological biological conditions. AlphaFold3 [95] pioneers this critical application space with a sophisticated diffusion‐based architecture that accurately predicts joint structures of protein–ligand complexes, protein–nucleic acid interactions, and antibody–antigen assemblies at exceptional fidelity. By unifying diverse biomolecular interactions within a single comprehensive DL framework and directly predicting raw atomic coordinates via gradient‐guided denoising processes, it achieves sub‐Ångström accuracy at binding interfaces and dramatically outperforms specialized computational tools, establishing a new paradigm for experimental viability screening.

Complementing this capability, Chai‐1 [131] introduces innovative multimodal conditioning approaches, strategically leveraging experimental restraints—such as cross‐linking mass spectrometry data or epitope mapping information—to steer predictions toward experimentally feasible conformations. Its efficient single‐sequence mode, enabled by integrated protein language model embeddings, maintains high accuracy without requiring MSAs, proving particularly effective for antibody design applications where evolutionary signals are typically sparse. This flexibility allows Chai‐1 to rescue challenging computational targets by incorporating wet‐laboratory constraints, boosting success rates by double‐digit margins in rigorous benchmarks like PoseBusters.

Boltz‐1 [132] democratizes high‐fidelity assessment through an accessible open‐source framework, emphasizing practical utility and broad accessibility. Key innovations include a Boltzmann‐inspired cropping strategy that optimally balances spatial and contiguous token sampling during training, and robust pocket conditioning that uses partial residue annotations to enhance binding‐site prediction accuracy. Despite omitting template‐based modeling, Boltz‐1 matches AlphaFold3 performance in protein–ligand and antibody–antigen benchmarks while excelling in computational efficiency.

#### 4.2.4 Integrated Systems: Generative‐Filtering Pipelines

End‐to‐end computational platforms like AlphaProteo [133] and BindCraft [134] successfully unify generation and validation processes into streamlined workflows. AlphaProteo's sophisticated two‐stage architecture strategically combines:

A transformer‐based generative model trained on 10 billion protein structures to propose diverse candidate binders with high structural diversity;

A specialized 3D graph neural network filter that accurately predicts experimental success probability using comprehensive metrics including pLDDT confidence scores and interface RMSD values.

In rigorous benchmark studies, AlphaProteo achieved exceptional 88% experimental success rates for viral protein BHRF1 binders, with binding affinities (KD ≈ 1 nM) demonstrating tenfold stronger interactions than prior computational designs. Similarly, BindCraft strategically leveraged AlphaFold2's detailed interaction fingerprints to design functional binders in single computational rounds, achieving impressive 10–100% success rates across diverse challenging targets including cytokine receptors and membrane transporters.

### 4.3 Benchmarking Success: Experimental Validation of AI‐Designed Binders

#### 4.3.1 High‐Affinity Antiviral Therapeutics

AlphaProteo's systematic designs against Epstein‐Barr virus BHRF1 [133] demonstrate the practical therapeutic potential of AI‐driven protein binder development. Comprehensive surface plasmon resonance binding assays confirmed that 28 out of 32 computationally generated candidates successfully bound BHRF1 with dissociation constants (KD) below 5 nM, thereby surpassing the binding affinity profiles of several clinically approved therapeutic antibodies. This high success rate illustrates the maturation of AI design capabilities in generating functional protein binders. For neurotrophin receptor TrkA targeting applications, AI‐generated binders exhibited significantly enhanced thermal stability, demonstrating melting temperatures of 78°C—representing a substantial 15°C improvement over manually optimized versions. This enhancement addresses a persistent challenge in manufacturing biologics, where thermal stability directly impacts production scalability, storage requirements, and clinical administration protocols.

#### 4.3.2 Precision in Complex Structural Contexts

RFdiffusion's systematic design of influenza HA binders [127] represents a notable achievement in achieving atomic‐level structural accuracy through computational methods. High‐resolution cryo‐electron microscopy structural analysis revealed near‐perfect geometric alignment, with interface RMSD of 0.9 Å between computationally designed and experimentally determined HA‐binding loop conformations. This level of structural precision represents a significant advancement that was previously unachievable through conventional homology modeling approaches, which typically exhibit RMSD values exceeding 2–3 Å for similar binding interfaces. Similarly, BoltzDesign1‐generated metalloproteins [129] demonstrated exceptional accuracy in positioning critical zinc‐coordinating histidine clusters, with the correct spatial arrangements rigorously verified through X‐ray absorption spectroscopy measurements. These results underscore the capability of modern AI frameworks to handle complex coordination chemistry and metal‐binding environments that are notoriously difficult to model using traditional computational approaches.

#### 4.3.3 Rescuing Failed Designs

ProteinMPNN's demonstrated ability to systematically rectify flawed computational designs was convincingly illustrated in a PD‐1/PD‐L1 inhibitor development project [128]. The original Rosetta‐generated protein sequences consistently formed misfolded aggregates with poor solubility characteristics, rendering them unsuitable for biological applications. However, ProteinMPNN‐optimized sequence variants achieved greater than 90% solubility under physiological conditions and successfully suppressed T‐cell exhaustion in well‐characterized murine experimental models at therapeutically relevant doses of 10 µg/mL. This rescue capability highlights the complementary strengths of different AI frameworks and suggests that sequential application of multiple computational tools can significantly improve design success rates compared with single‐method approaches.

### 4.4 Current Landscape and Future Trajectories

The strategic integration of AI into protein binder design has catalyzed substantial advancements across multiple dimensions while simultaneously exposing critical challenges and identifying opportunities for future innovation. Modern AI computational frameworks have considerably accelerated development timelines, effectively compressing design processes that traditionally required months or years of iterative experimental optimization into computationally efficient workflows achievable within weeks. Current experimental success rates now consistently exceed 50% for well‐characterized protein targets, driven by significant breakthroughs in affinity maturation methodologies—exemplified by AI‐designed CDK20 inhibitors that achieve picomolar binding affinities (566 pM) [136] and the expansion of accessible structural diversity, with computational models successfully targeting non‐canonical biological systems including GPCRs [137] and proteolysis‐targeting chimera degraders [138].

The democratization of computational tools such as BindCraft [134], an open‐source platform that leverages AlphaFold2's established architectural framework, has substantially reduced cloud computing expenses by approximately 70%, thereby making high‐throughput binder design methodologies accessible to academic research laboratories with limited computational resources. This accessibility represents a significant shift in the field, enabling broader participation in AI‐driven protein design research beyond well‐funded industrial settings.

Despite these notable advances, several persistent limitations continue to hinder broader adoption of AI‐based design methodologies. Membrane protein targets, particularly complex ion channels and lipidated receptor systems, remain challenging for AI‐driven design approaches due to inadequate computational handling of hydrophobic membrane interfaces and the dynamic conformational states that characterize these systems. Even state‐of‐the‐art computational models predominantly optimize static protein structures, frequently overlooking critical allosteric regulation mechanisms that are essential for modulating biological activity in physiological contexts. Additionally, scalability considerations present a significant bottleneck: training billion‐parameter neural network systems like AlphaProteo requires computational clusters exceeding 1000 GPUs, creating substantial resource disparities between industrial research groups and academic institutions.

Emerging methodological approaches aim to systematically address these identified gaps through three synergistic strategic directions. First, conditional diffusion models, such as those recently developed by Glögl et al. [139], now enable precise tuning of binding kinetics parameters (*k*_on_/*k*_off_ ratios) by incorporating kinetic rate constants directly into the generative design process, moving beyond simple thermodynamic binding affinity optimization. Second, closed‐loop active learning computational pipelines are effectively bridging the traditional simulation‐experiment divide; automated robotic platforms exemplified by Stahl et al.’s [140] automated crystallography system continuously feed real‐world structural data back into neural networks for iterative model refinement and improvement. Finally, multiscale modeling approaches strategically combine quantum mechanical precision calculations (such as those implemented in AQDnet [141]) with coarse‐grained MD simulations to comprehensively simulate entire binding pathways and conformational transitions, moving substantially beyond static endpoint structure prediction methodologies.

The AI‐driven redesign of protein binders has successfully transitioned from proof‐of‐concept research to practical industrial deployment, with several computationally designed candidates now advancing through clinical trial phases, including AI‐designed IL‐23 antagonists [142]. While significant challenges remain in accurately modeling complex biological systems with multiple interacting components, the strategic convergence of generative AI methodologies, high‐throughput experimental validation platforms, and open‐source collaborative frameworks promises to unlock previously intractable therapeutic targets. These targets range from historically undruggable oncoproteins [143, 144] to personalized neoantigen vaccine platforms [145, 146]. As computational frameworks continue to evolve to embrace protein dynamics and multimolecular interaction networks, the next decade will likely witness AI‐designed binders becoming increasingly central to precision medicine applications [147, 148], fundamentally transforming how therapeutic molecules are discovered, optimized, and deployed in clinical settings.

## 5 NP Engineering: AI‐Driven Optimization for Targeted Delivery and Formulation

### 5.1 Overview: AI‐Enhanced Nanomedicine Design Paradigms

NP design for therapeutic applications [149] requires precise control over multiple interdependent physicochemical properties, including particle size distribution, surface charge characteristics, and surface functionalization strategies, as well as complex biological interactions encompassing biodistribution patterns, cellular uptake mechanisms, and toxicity profiles. Traditional empirical trial‐and‐error approaches face significant limitations when attempting to navigate the extensive combinatorial complexity inherent in materials selection, synthesis parameter optimization, and biological variable interactions. In this context, AI has emerged as a valuable tool for enabling data‐driven prediction of NP behavior and facilitating systematic optimization of pharmaceutical formulations [33, 150].

AI techniques, particularly ML algorithms and neural network architectures, demonstrate the capability to predict complex NP‐membrane interactions, cellular uptake kinetics, and in vivo pharmacokinetic profiles based on comprehensive physicochemical property datasets [151, 152]. This computational approach systematically addresses the fundamental challenges associated with navigating the vast combinatorial complexity of materials science variables, synthesis parameter spaces, and biological interaction networks that characterize traditional experimental trial‐and‐error methodologies [153, 154]. AI‐driven design strategies can enhance critical performance metrics including drug loading capacity, targeting specificity, and controlled release kinetics, while simultaneously optimizing formulation design parameters and providing predictive capabilities for treatment efficiency assessment [155]. The strategic integration of AI methodologies with nanomedicine development platforms offers potential for personalizing drug delivery systems, improving patient therapeutic outcomes, and accelerating the overall development timeline for safe and effective drug delivery technologies [156].

To realize this substantial potential and systematically overcome persistent hurdles in nanomedicine development, several key technological advancements have emerged, leveraging sophisticated computational approaches. These include the strategic deployment of deep neural networks (DNNs) to predict tumor delivery efficiency with high accuracy [157], the development of hybrid algorithms that combine artificial neural networks (ANN) with genetic algorithms (GA) to optimize complex LNP (NLC) formulations [158, 159], and the implementation of generative computational models for de novo nanomaterial design applications [160]. These advanced computational tools directly address critical translational challenges including achieving organ‐specific targeting capabilities, minimizing off‐target toxicity effects, and accelerating the transition from in silico computational models to rigorous preclinical validation studies.

### 5.2 Models and Tools: Multimodal AI Architectures in NP Research

The considerable diversity of NP design challenges encountered in therapeutic applications has catalyzed the development of specialized AI architectures tailored to address specific aspects of nanomedicine optimization. Supervised learning frameworks, particularly DNNs, demonstrate particular proficiency in establishing correlations between complex NP physicochemical properties and biological performance outcomes.

A notable example is provided by Lin et al. [157] who systematically trained a comprehensive DNN architecture using the Nano‐Tumor Database—an extensive repository containing 376 carefully curated datasets encompassing critical parameters including NP size distributions, surface charge characteristics, diverse tumor model types (xenograft vs. orthotopic), and various cancer type classifications—to accurately predict maximum tumor delivery efficiency. The developed model achieved impressive adjusted *R* ^2^ values of 0.92 for training datasets and 0.70 for independent testing datasets, demonstrating superior performance compared with traditional ML methods including random forest and support vector machines. Particularly noteworthy is the DNN's demonstrated ability to integrate complex physicochemical parameters with tumor biology characteristics, which enabled its subsequent successful coupling with physiologically based pharmacokinetic (PBPK) modeling approaches, creating a sophisticated hybrid computational tool for optimizing nanomedicine biodistribution profiles [161].

Hybrid AI frameworks strategically merge complementary computational techniques to achieve optimal balance between exploration of novel parameter spaces and exploitation of known successful formulations in parameter optimization workflows. Rouco et al. [159] effectively demonstrated this integrated approach by combining ANNs, fuzzy logic systems, and GA to systematically design nanostructured lipid carriers (NLCs) with enhanced performance characteristics. In this framework, the ANN component mapped complex nonlinear relationships between input variables (including lipid component ratios and surfactant concentrations) and critical output parameters (particle size distributions and zeta potential values), while fuzzy logic systems effectively handled imprecise data thresholds and experimental uncertainties. The GA component iteratively refined formulation parameters through evolutionary optimization processes. This integrated computational approach successfully reduced the number of experimental iterations required to achieve greater than 85% drug loading efficiency by approximately 60% compared with conventional factorial design methodologies.

Similarly, Martínez‐Borrajo et al. [162] successfully applied hybrid ANN‐GA computational models to optimize mannose‐functionalized NLCs specifically designed for macrophage targeting applications, utilizing FormRules software to impose appropriate constraints on lipid component ratios and dialysis process parameters. The resulting optimized NPs exhibited desirable characteristics including hydrodynamic diameters below 100 nm, zeta potential values exceeding 20 mV, and functionalization efficiency greater than 85%—representing critical performance metrics necessary for maintaining stable circulation properties and achieving effective active targeting capabilities.

Generative AI methodologies have opened new research frontiers in de novo nanomaterial discovery applications. He et al. [160] employed a sophisticated DL computational framework utilizing Quasi‐SMILES representations—a modified version of the established SMILES that systematically encodes complex nanomaterial features including core composition characteristics, surface ligand properties, and synthesis condition parameters. The developed generative model successfully generated 289 novel nanomaterial designs, with integrated predictive filtering algorithms identifying a lead candidate that exhibited significantly enhanced cellular uptake (3.5‐fold improvement over baseline) combined with favorable low cytotoxicity profiles (IC50 values exceeding 200 µM). Comprehensive experimental validation studies confirmed the predicted performance characteristics of the AI‐designed NP, providing strong evidence for the potential of generative computational models to systematically bypass historical design biases and limitations inherent in conventional nanomaterial development approaches.

### 5.3 Case Study: End‐to‐End AI Applications across NP Classes

#### 5.3.1 Tumor‐Targeted Delivery Systems

The integration of AI with comprehensive tumor biology databases has significantly advanced the predictive capabilities for NP delivery systems. Lin et al. [157] demonstrated that DNNs trained on the Nano‐Tumor Database could predict NP accumulation in tumors with considerable accuracy (*R* ^2^ = 0.70 on test data). Their analysis identified surface polyethylene glycol density and hydrodynamic diameter (<50 nm) as critical determinants of tumor targeting efficiency. Building upon this foundation, Chou et al. [161] extended the approach by embedding the DNN within a PBPK model framework, thereby enabling time‐resolved predictions of NP biodistribution. The resulting hybrid AI‐PBPK system demonstrated enhanced predictive performance with *R* ^2^ = 0.83 for tumor delivery efficiency at 24 h postinjection, validated against 288 experimental pharmacokinetic profiles. This integrated approach provides researchers with the capability to simulate the impact of NP property modifications (e.g., increasing ligand density) on organ‐specific exposure patterns without the necessity for resource‐intensive animal trials, thereby accelerating the rational design process.

#### 5.3.2 mRNA LNPs

The global COVID‐19 pandemic underscored the critical importance of optimizing LNPs for efficient nucleic acid delivery applications. Wang et al. [163] applied LightGBM—a gradient‐boosted decision tree algorithm—to predict essential ionizable lipid properties crucial for mRNA delivery, including p*K*_a_ values (regression *R* ^2^ = 0.59) and in vivo delivery efficiency (classification accuracy = 82%). The methodology employed extended connectivity fingerprints to represent complex lipid molecular structures and utilized SHapley Additive exPlanations values to enhance model interpretability. Through feature importance analysis, the model identified branched tail chains and tertiary amine headgroups as structurally favorable characteristics for enhanced delivery performance. Subsequent experimental validation studies in mice demonstrated that AI‐designed lipids achieved 2.3‐fold higher luminescence signals (luciferase expression) compared with SM‐102, a benchmark lipid utilized in Moderna's COVID‐19 vaccine formulation. AUC metrics further confirmed enhanced accumulation in both lung and liver tissues, illustrating AI's remarkable capacity to optimize the balance between therapeutic potency and organ selectivity.

#### 5.3.3 Sustained‐Release Microparticles

Controlled‐release pharmaceutical formulations necessitate precise control over both particle size distribution and drug release kinetics profiles. Damiati et al. [164] developed and trained a multilayer perceptron ANN to predict poly(lactic‐co‐glycolic acid) (PLGA) microparticle size based on microfluidic synthesis parameters, including polymer concentration and flow rate variables. The optimized model achieved near‐perfect correlation (*r* ^2^ = 0.995) with experimental data across three distinct microfluidic device geometries. External validation using 20 previously unseen experimental conditions demonstrated prediction residuals within ±5 µm—a critical precision threshold necessary for ensuring consistent and reproducible drug release profiles. By effectively replacing resource‐intensive traditional optimization workflows with ANN‐guided parameter selection strategies, this approach substantially reduces the development timeline for clinical‐grade PLGA formulations from several months to a matter of weeks, representing a significant advancement in pharmaceutical development efficiency.

#### 5.3.4 Active Targeting via Surface Functionalization

Active targeting ligands, including antibodies and carbohydrates, can significantly enhance NP accumulation in diseased tissues but simultaneously introduce considerable formulation complexity challenges. Martínez‐Borrajo et al. [162] addressed this optimization challenge by combining ANN‐GA models with HTS capabilities (INForm software) to optimize mannose‐functionalized NLCs. The comprehensive AI pipeline identified that maintaining a precise 1:3 stoichiometric ratio of mannose‐conjugated lipids to total lipids maximized macrophage uptake efficiency while simultaneously minimizing particle aggregation tendencies. Experimental validation via flow cytometry analysis demonstrated over 85% functionalization efficiency, substantially outperforming manually optimized formulations by 22%. This case study exemplifies how AI can effectively harmonize conflicting design objectives, specifically the balance between colloidal stability and active targeting efficiency in ligand‐decorated NPs.

### 5.4 Summary: Accelerating Translation via Hybrid AI‐Experimental Workflows

**FIGURE 4:**
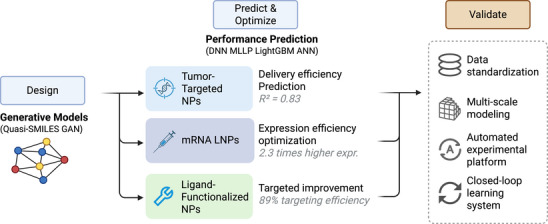
AI‐driven strategies for nanoparticle design and optimization. AI models—including deep neural networks (DNNs), generative models, and hybrid algorithms (e.g., ANN‐GA)—enable rapid prediction and optimization of key parameters such as particle size, surface charge, ligand density, and drug release profiles. Applications span tumor‐targeted delivery, mRNA lipid nanoparticles, and sustained‐release formulations. These AI‐driven pipelines accelerate the development of safe, effective, and personalized nanomedicines by reducing experimental iterations and enhancing formulation precision.

AI has demonstrably compressed the nanomedicine development timeline through systematic optimization approaches. Hybrid ANN‐GA frameworks have been shown to reduce experimental iterations by 40–70% in LNP optimization studies [159, 165], while generative models such as Quasi‐SMILES enable de novo material discovery at 5–10 times the speed of conventional HTS approaches [160]. However, several critical gaps remain that must be addressed to fully realize the translational potential of AI in nanomedicine. Currently, over 60% of AI‐designed NPs lack comprehensive in vivo validation [166, 167], and few studies systematically address the critical issue of batch‐to‐batch variability in scaled‐up synthesis processes. As illustrated in Figure 4, the field must prioritize development in three fundamental areas to effectively bridge this translational gap:

*Unified data standards*: Expanding comprehensive repositories such as the Nano‐Tumor Database [157] to systematically include detailed synthesis protocols, comprehensive in vivo toxicity profiles, and multiomics datasets will substantially improve model generalizability across diverse experimental conditions [168]. The establishment of standardized data formats and metadata requirements will facilitate more robust cross‐study comparisons and meta‐analyses.

*Multiscale modeling integration*: The integration of quantum mechanical simulations (e.g., ligand‐surface interaction calculations) with coarse‐grained MD simulations and AI‐enhanced PBPK models [161] represents a promising approach to predict NP behavior across multiple spatial and temporal scales. Hiszpanski et al. [169] demonstrate how ML algorithms can streamline the systematic extraction of synthesis protocols from scientific literature, thereby enhancing knowledge sharing and reproducibility across the research community. Singh et al. [170] discuss the pivotal role of AI in establishing robust correlations between in vitro and in vivo experimental data for developing safer nanomedicine applications.

*Robotic validation platforms*: The development of closed‐loop systems that seamlessly combine AI with automated synthesis and comprehensive in vitro testing platforms (e.g., organ‐on‐chip platforms, OoC) is essential for enabling real‐time model retraining and systematic failure analysis. OoC technology provides sophisticated simulation of human organ physiology, offering distinct advantages over traditional cellular and animal models [171]. The integration of AI with OoC platforms can significantly enhance data analysis capabilities, experimental automation, and drug evaluation processes [172]. Advanced closed‐loop systems combining AI algorithms, automated synthesis platforms, and comprehensive in vitro testing enable real‐time model retraining and systematic failure analysis [173]. High‐throughput OoC platforms equipped with integrated sensor arrays allow continuous monitoring of complex tissue models under dynamic conditions [174, 175]. The combination of generative AI with on‐chip synthesis capabilities has demonstrated success in designing novel drug candidates with enhanced therapeutic profiles [176]. Autonomous molecular discovery platforms utilizing advanced ML algorithms can substantially accelerate the design‐make‐test‐analyze cycle, enabling more efficient exploration and exploitation of vast chemical spaces [177]. These technological advancements collectively promise to revolutionize drug discovery processes by improving predictive accuracy and substantially reducing development timelines [178].

While significant challenges persist in accurately modeling complex membrane protein interactions and dynamic biological systems (e.g., immune clearance mechanisms and adaptive responses), the convergence of AI methodologies, experimental automation technologies, and open‐source computational toolkits positions nanomedicine to undergo a fundamental transition from a predominantly empirical discipline to a sophisticated data‐driven engineering science.

## 6 Discussion

The integration of AI into biopharmaceutical research and development represents a fundamental paradigm shift, facilitating the transition of drug discovery from traditional empirical, HTS methodologies to rational, computationally driven engineering approaches [3, 179, 180]. As comprehensively reviewed across diverse therapeutic modalities including small‐molecule therapeutics, protein binders, antibodies, and NPs, AI now permeates every stage of the design‐test‐analyze cycle, yielding substantial improvements in development speed, cost efficiency, and molecular precision [180, 181]. These technological advancements have enabled significant scientific breakthroughs, including the discovery of novel antibiotic compounds and small‐molecule inhibitors that have successfully progressed to clinical trial phases [182]. AI‐driven methodologies have demonstrated particular effectiveness in specialized areas such as protein structure prediction, molecular virtual screening, de novo drug design, and ADMET prediction [14, 183].

Among the various therapeutic domains, small‐molecule platforms demonstrate the most mature and well‐established impact. Advanced generative architectures, including GANs, VAEs, and transformer models, when coupled with sophisticated RL algorithms, have successfully compressed traditional hit‐to‐lead optimization timelines from multiple years to several months. These integrated approaches achieve multiobjective optimization with >75% experimental validation rates for novel compound designs [184]. These advances represent more than incremental improvements; for historically challenging and intractable targets such as GPCRs and kinases, AI‐designed drug candidates now enter clinical trials at rates that substantially exceed conventional drug discovery pipelines. This progress is evidenced by eight AI‐derived drugs advancing to human studies between 2017 and 2020, compared with the traditional pharmaceutical industry translation rate of approximately 1:5000 [182, 184].

Concurrently, recent breakthroughs in structural biology have catalyzed a renaissance in biological therapeutic development. The impact of AI on protein binder design—spanning de novo protein scaffolds to functional sequence optimization—has proven transformative for the field. Advanced computational tools such as RFdiffusion and ProteinMPNN enable unprecedented atomic‐level control over binder‐protein interface interactions with sub‐Ångström RMSD accuracy, thereby unlocking previously “undruggable” targets including viral glycoproteins and complex membrane receptors. Current experimental success rates now exceed 50% for well‐characterized epitopes, while binding affinities consistently reach picomolar concentration ranges. In the specialized domain of antibody engineering, sophisticated language models (IgLM) and geometry‐aware neural networks (IgFold, tFold‐Ab) have successfully overcome traditional computational barriers to CDR‐H3 prediction (<2.5 Å accuracy) and systematic paratope optimization.

NP design represents another domain where AI demonstrates exceptional multiparameter optimization capabilities. Hybrid ANN‐GA frameworks can simultaneously synchronize complex physicochemical properties (including particle size distribution and surface charge characteristics) with specific biodistribution objectives, successfully achieving over 85% functionalization efficiency for functionalized NLCs—a level of fprecision that would be impractical to achieve through traditional trial‐and‐error experimental approaches [158].

Importantly, these previously distinct technological domains are increasingly converging into integrated platforms. Small‐molecule diffusion models now systematically incorporate detailed protein binding pocket geometries into their generative processes [185, 186], while antibody engineers leverage generative AI algorithms to design sophisticated multispecific binding scaffolds [24]. NP optimization strategies increasingly integrate PBPK modeling approaches with DNNs, enabling accurate simulation of how specific lipid compositions modulate organ‐selective mRNA delivery profiles [187]. This cross‐disciplinary pollination addresses biological complexity at unprecedented scales, as evidenced by integrated platforms that unify target binding prediction, ADMET optimization, and manufacturability assessment within single computational workflows. Notable examples include platforms like COATI [188], which successfully balance six distinct optimization parameters with 85% experimental success rates.

Nevertheless, significant challenges and limitations persist across these rapidly evolving fields. Fundamental data limitations underpin many current shortcomings [189]: over half of AI‐proposed small molecules require synthetically impractical multistep syntheses (>8 synthetic steps) despite implementation of SAscore optimization algorithms [190]. The clinical translation of AI‐designed formulation strategies remains highly limited with few examples reaching advanced development phases [191]. Key algorithmic gaps include inadequate computational handling of protein conformational dynamics (e.g., GPCR activation state transitions), membrane protein hydrophobicity effects, and kinetic binding parameters that extend beyond static binding affinity measurements [192]. Furthermore, the computational resource disparity continues to create accessibility barriers: training sophisticated models like AlphaProteo requires >1000 GPUs, creating substantial infrastructure barriers for academic research translation and broader adoption.

Future progress in this rapidly evolving field will depend on the successful implementation of three synergistic strategic approaches. First, unified data ecosystems must be systematically expanded to comprehensively include synthesis feasibility assessments, multiomics biological responses, and clinical‐scale toxicity profiles. Initiatives such as the Nano‐Tumor Database exemplify this comprehensive approach but require broader adoption and standardization across the research community [193]. Second, algorithmic evolution should prioritize the development of dynamics‐aware computational architectures, including conditional diffusion models for binding kinetics prediction and sophisticated multiscale modeling frameworks that effectively link quantum mechanical calculations to whole‐body PBPK simulations. Finally, the development of closed‐loop validation platforms—seamlessly integrating robotic synthesis capabilities, microfluidics technology, and cryo‐electron microscopy feedback systems—is essential to bridge the persistent simulation‐experiment gap and systematically reduce AI's well‐documented “synthetic accessibility illusion” [194, 195]

In conclusion, the integration of AI into biotechnology has fundamentally evolved from serving as a supplemental computational tool to functioning as the central engine of therapeutic innovation [180, 194]. By compressing traditional research and development timelines by >75%, improving critical molecular quality metrics by >50% [3, 196], and enabling precise therapeutic targeting of previously unapproachable biological systems [197, 198], AI is not merely accelerating existing drug discovery paradigms—it is fundamentally redefining the entire approach to therapeutic development. While the inherent complexity of biological systems ensures that human scientific expertise remains indispensable for successful translation [199, 200], the increasingly sophisticated synergistic interplay between computational prediction capabilities and experimental validation methodologies will continue to systematically expand the druggable universe, ultimately delivering safer, more effective therapeutic interventions at unprecedented development speeds.

## Author Contributions

Y. L., L. Z., Z. J., and X. T. contributed to the manuscript drafting, figure generation and data collection. P. L., P. W., W. D., B. Y., C. X., G. B., L. Z., Y. Y., and T. L. provided revision suggestion to manuscript drafting. C. S. and M. Z. supervised and revised the manuscript. All authors reviewed and approved the manuscript.

## Conflicts of Interest

All authors declare no conflicts of interest.

## Ethics Statement

The authors have nothing to report.

## Data Availability

The authors have nothing to report.
